# Cost-effectiveness of phosphate binders among patients with chronic kidney disease not yet on dialysis: a long way to go

**DOI:** 10.1186/s12882-016-0286-7

**Published:** 2016-07-08

**Authors:** Rana Rizk

**Affiliations:** Department of Health Services Research, CAPHRI School of Public Health and Primary Care, Maastricht University, 6200 MD Maastricht, The Netherlands

**Keywords:** Chronic kidney disease, Phosphate binders, Hyperphosphatemia, Cost-effectiveness analysis

## Abstract

Hyperphosphatemia management is integral to the management of patients with chronic kidney disease. This mineral abnormality is associated with greater costs, but so is its management, especially with the use novel phosphate binders. The economic evaluation of these pharmaceutical agents is increasingly needed to provide evidence for value of money spent and inform resource allocation. Recently, Nguyen et al. explored the economical attractiveness of Sevelamer relative to Calcium Carbonate among patients with chronic kidney disease not yet on dialysis and concluded that the former was cost-effective. The current commentary discusses the results of this analysis and sheds light on the methodological challenges of economic evaluations in this field.

## Background

Hyperphosphatemia is consistently and independently associated with increased morbidity and mortality among end stage renal disease patients [1–3], and results in financial burdens for health systems [4]. Although this issue was explored to a lesser extent among patients with chronic kidney disease (CKD), results from large studies suggest an independent association between elevated serum phosphorus levels and increased patient mortality risk [5, 6]. The association between hyperphosphatemia and progression to dialysis initiation has also been suggested. However, this association was inconsistent across studies [7] and was rendered not statistically significant when potential confounders were accounted for in the analysis [6]. Despite this, serum phosphorus management in CKD patients has gained increasing importance in contemporary nephrology practice, and tight targets for serum phosphorus levels were set for these patients, although, up-till-now, we still lack conclusive evidence, ie prospective interventional studies, to demonstrate that reduction in serum phosphate improves CKD patient outcomes.

Concerns about the high cost of hyperphosphatemia’s consequences are offset by concerns about hyperphosphatemia’s high treatment cost [8], especially with the novel pharmaceuticals, ie non-calcium-based phosphate binders. Within this scope, economic evaluations *-studies providing evidence for value of money spent*- are now being increasingly used by public health decision makers to guide the allocation of scarce resources [9].

## Main text

The cost-effectiveness of phosphate binders was first addressed among hemodialysis patients, where due to many factors, the management of serum phosphorus is a continuous challenge. Since 2005, multiple full economic evaluations were published in this regards. In 2016, the comparative cost-effectiveness of these agents was systematically explored by Rizk et al. [10], where in view of the suboptimal quality and inconsistent results of included studies, robust conclusions could not be generated. The authors of the review suggested that calcium-based binders- especially Calcium Acetate was the most economically attractive therapy, in first-line and sequential use, in prevalent patients, and that Lanthanum Carbonate might provide good value for money, as second-line therapy, in incident patients. In that review, the incremental cost-utility ratio of Sevelamer relative to Calcium Carbonate ranged between US$36,803 and US$157,760 per quality adjusted life year (QALY) gained for first-line use among prevalent patients. Relative to the same comparator, Sevelamer was borderline cost-effective among incident patients (US$47,153).

So far, the comparative cost-utility of phosphate binders among renal patients not yet on dialysis was only explored in two studies. The first by Vegter et al. [11] concluded that second-line Lanthanum Carbonate dominated -*less costly and more effective-* calcium-based binders. Cost savings were mainly attributed to the delayed CKD progression and dialysis initiation. The second study by Thompson et al. [12] concluded that first-line Sevelamer was cost-effective compared with Calcium Carbonate (incremental cost of £23,878 per QALY gained (US$36,475)). However, in this study, the results were most sensitive to alternative assumptions regarding the impact of Sevelamer on dialysis initiation. Both studies [11, 12] adopted the perspective of the National Health Service in the United Kingdom and used a lifelong Markov model. Accordingly, one would suggest that the cost-effectiveness of non-calcium based binders is mainly driven by their *perceived* effect on delaying the treatment by dialysis.

This issue of BMC Nephrology includes the third full economic evaluation of phosphate binders among patients with CKD not yet on dialysis. The article by Nguyen et al. [13] assessed the life-time incremental cost-utility of first-line Sevelamer relative to Calcium Carbonate from the perspective of a third party payer in Singapore, using a Markov model. This study was funded by Sanofi-Aventis (Singapore). The authors concluded that Sevelamer produces an incremental cost-utility ratio of S$51,756 (US$38,500) per QALY gained relative to Calcium Carbonate.

In contrast to the above-mentioned two studies, where country-specific clinical evidence was readily available for the analysis, Nguyen et al. [13] employed a multitude of clinical effectiveness sources to construct their model, some of which were extracted from divergent patient population groups. In order to circumvent limitations of available Singapore-specific data, especially survival outcomes (mortality risks among Singaporean CKD and dialysis patients), the authors used age-specific mortality risks from Singapore life-tables, mortality hazard ratios for CKD patients based on a large Taiwanese cohort study and adjusted age-specific mortality risks from the United States Renal Data System. However, other key parameters used in the model, such as the probabilities for transitioning to dialysis or the needed dose of Sevelamer to reach a certain level of effectiveness were directly extrapolated from divergent patient groups [14] to the study’s population. This issue must be revisited and further research in this regards must be conducted in order to inform public health decision making, especially that the sensitivity analyses showed that Sevelamer became less economically attractive with higher dialysis costs and at higher Sevelamer doses. Finding readily available country-specific data for model inputs or information to adjust data from other countries is a consistent challenge across model-based economic evaluation studies, especially those conducted in low and middle income countries. This was repeatedly faced by authors from these countries in the field of phosphate-binder related research among renal patients [10].

Similarly, although Nguyen et al. [13] presented thorough details of the data used for constructing the model, caution must be taken when directly extrapolating their results to be used in other countries. In fact, the incremental cost-utility ratio of Sevelamer versus Calcium Carbonate was most sensitive to changes in the cost of the former. This agent was shown to be no longer cost-effective at the price of S$1.69/g (US$1.26/g). Given the changes in the costs of therapeutic agents across countries [15, 16], the direct transferability of these results is questioned [16]. This issue was also pinpointed in other areas of economic evaluations of health care interventions [17]. In fact, strong evidence points out the challenging and complex task of the transferability of economic evaluation data across countries, and that transferability requires a minimum of country-specific substitution of practice pattern data, in addition to unit cost data [16].

On the other hand, Grima et al. [18] presented the case for excluding dialysis costs in economic evaluations of interventions that increase survival, such as phosphate binders. The authors argued that due to the high cost of dialysis, the inclusion of its costs in the analysis of any life-extending intervention eliminates the possibility of obtaining a favorable cost-effectiveness ratio, regardless of the clinical benefits of the intervention. According to the authors, this might deny renal patients access to interventions that are initially cost-effective. Assessing the impact of excluding future dialysis costs in the sensitivity analyses would have presented a more realistic look at the cost-effectiveness of Sevelamer. Moreover, non-calcium based phosphate binders seem to be more economically attractive when used as a second line therapy among dialysis and non-dialysis patients [10, 11]. In depth exploration of this issue would have been interesting in order to inform decision making, especially in countries with limited health resources.

This study was funded by a pharmaceutical company. Previous systematic reviews of the literature [19, 20] found that pharmaco-economic studies sponsored by the industry were more likely to favor the sponsor’s product. This was also the case of publications on phosphate binders for hyperphosphatemia management [10]. Sponsor bias (specifically in trial-based economic evaluations) was thoroughly discussed by Evers et al. [21] and solutions to overcome it, beyond disclosure of the financial conflict of interest and a rigorous peer-review process, included maintenance of good methodological standards. This highlights the pressing need for stringent guidelines governing the conduct and publication of economic evaluations.

Finally, in contrast to the case of hemodialysis patients, where various evidence-based practice guidelines agree on actively managing hyperphosphatemia, this issue is less clear among patients not receiving dialysis. While the Kidney Disease: Improving Global Outcomes (KDIGO) guidelines [22] recommend maintaining serum phosphate levels “within the normal range” starting stage 3 or 4 of the disease, the United Kingdom National Institute for Health and Clinical Excellence (NICE) guidelines [23] suggest that serum phosphate should be monitored routinely only in stages 4, 5, and 5d. Nevertheless, in all of the above-mentioned cases, the guidelines were not based on definitive evidence on the beneficial effect of lowering phosphate levels in early or late CKD.

## Conclusion

In conclusion, evaluating the outcomes of the implementation of renal guidelines pertaining to serum phosphorus management in terms of reduced morbidity, mortality and delayed dialysis initiation is becoming imperative. This would pave the way for a more rationale evaluation of the cost-effectiveness of interventions in this regards. Until then, employing borderline cost-effective phosphate binders to achieve tight serum phosphorus levels, where supportive conclusive evidence surrounding the clinical or financial benefits is lacking, is to be discussed in the light of the country-specific health priorities and budgetary limitations.

## Abbreviations

CKD, chronic kidney disease; KDIGO, kidney disease: improving global outcomes; NICE, National Institute for Health and Clinical Excellence; QALY, quality adjusted life year
